# Evaluation of AJCC Nodal Staging for Intraductal Papillary Mucinous Neoplasm-Derived Pancreatic Ductal Adenocarcinoma

**DOI:** 10.1245/s10434-024-16055-5

**Published:** 2024-09-16

**Authors:** Joseph R. Habib, Ingmar F. Rompen, Ammar A. Javed, Anthony M. Sorrentino, Mansour E. Riachi, Wenqing Cao, Marc. G. Besselink, I. Quintus Molenaar, Jin He, Christopher L. Wolfgang, Lois A. Daamen

**Affiliations:** 1 Department of Surgery, New York University Langone Health, New York, NY USA; 2 Department of Surgery, Regional Academic Cancer Center Utrecht, UMC Utrecht Cancer Center and St. Antonius Hospital Nieuwegein, Utrecht, The Netherlands; 3 Department of Surgery, Amsterdam UMC, University of Amsterdam, Amsterdam, The Netherlands; 4 Cancer Center Amsterdam, Amsterdam, The Netherlands; 5 Department of Pathology, New York University Langone Health, New York, NY USA; 6 Department of Surgery, Johns Hopkins Hospital, Baltimore, MD USA; 7 Division of Imaging and Oncology, University Medical Center Utrecht, Utrecht, The Netherlands

## Abstract

**Background:**

The American Joint Committee on Cancer (AJCC) eighth edition is based on pancreatic intraepithelial neoplasia-derived pancreatic ductal adenocarcinoma (PDAC), a biologically distinct entity from intraductal papillary mucinous neoplasm (IPMN)-derived pancreatic cancer. The role of nodal disease and the AJCC’s prognostic utility for IPMN-derived pancreatic cancer are unclear. This study aimed to evaluate the prognostic role of nodal disease and the AJCC eighth-edition N-staging for IPMN-derived pancreatic cancer.

**Methods:**

Upfront-surgery patients with IPMN-derived PDAC from four centers were stratified according to the AJCC eighth-edition N stage. Disease characteristics were compared using descriptive statistics, and both overall survival (OS) and recurrence-free survival (RFS) were evaluated using log-rank tests. Multivariable Cox regression was performed to determine the prognostic value of N stage for OS, presented as hazard ratios with 95 % confidence intervals (95 % CIs). A lowest *p* value log-rank statistic was used to derive the optimal cutoff for node-positive disease.

**Results:**

For 360 patients, advanced N stage was associated with worse T stage, grade, tubular histology, and perineural and lymphovascular invasion (all *p* < 0.05). The median OS was 98.3 months (95 % CI 82.8–122.0 months) for N0 disease, 27.8 months (95 % CI 24.4–41.7 months) for N1 disease, and 18.1 months (95 % CI 16.2–25.9 months) for N2 disease (*p* < 0.001). The AJCC N stage was validated and associated with worse OS (N1 [HR 1.64; range, 1.05–2.57], N2 [HR2.42; range, 1.48–3.96]) and RFS (N1 [HR 1.81; range, 1.23–2.68], N2 [HR 3.72; range, 2.40–5.77]). The optimal cutoff for positive nodes was five nodes.

**Conclusion:**

The AJCC eighth-edition N-staging is valid and prognostic for both OS and RFS in IPMN-derived PDAC.

**Supplementary Information:**

The online version contains supplementary material available at 10.1245/s10434-024-16055-5.

Pancreatic ductal adenocarcinoma (PDAC) is associated with meager survival due to its tendency for local and systemic disease progression, even after surgical resection of the primary tumor.^1,2^ Multiple clinicopathologic factors such as tumor size, nodal disease, metastatic disease, grade of differentiation, and perineural and lymphovascular invasion have been associated with oncologic outcomes in PDAC.^3–8^ The presence of nodal disease in particular has been consistently and strongly implicated in systemic disease recurrence and poorer survival.^7,9–11^

Currently, the eighth edition of the American Joint Committee on Cancer (AJCC) staging system for pancreas cancer recommends defining nodal disease into three groups: N0 (0 positive nodes), N1 (1–3 positive nodes), and N2 (≥4 positive nodes).^12^ This is a marked change from the seventh edition, which split N stage into a binary classification of the presence (N1) or absence (N0) of nodal regional lymph node metastasis.^13^ Numerous studies have validated the AJCC eighth-edition classification for PDAC, demonstrating respectable prognostication performance with varying new recommendations to improve discrimination.^3,14–16^

Existing classifications and validations are largely based on pancreatic intraepithelial neoplasia (PanIN)-derived PDAC, which currently is accepted to be biologically distinct from intraductal papillary mucinous neoplasia (IPMN)-derived PDAC.^17^ Furthermore, survival outcomes are significantly different between these two distinct entities.^18,19^ Like PanIN-derived PDAC, the presence of nodal disease is a robust predictor of disease recurrence and survival in IPMN-derived PDAC.^20–22^ In fact, one recent multicenter study reported that the AJCC seventh edition may be more applicable to IPMN-derived PDAC because significant discrimination was not observed in patients with node-positive disease.^23^

This multi-institutional study aimed to comprehensively investigate the role of nodal disease in IPMN-derived PDAC and to evaluate the prognostic discrimination of the currently used eighth edition of the AJCC N stage classification for PanIN-derived PDAC in IPMN-derived PDAC.

## Methods

The study enrolled patients who underwent surgical resection for pathologically confirmed IPMN-derived PDAC at four high-volume international centers including Amsterdam University Medical Center (between 2013 and 2021), Johns Hopkins Hospital (between 1995 and 2021), New York University Langone Health (between 2014 and 2021), and the Regional Academic Cancer Center Utrecht (between 2005 and 2019). The exclusion criteria ruled out concomitant PanIN-derived PDAC arising separately from an adjacent IPMN, receipt of neoadjuvant therapy, gross positive resection margin, missing N stage, metastatic disease at diagnosis, and 90-day postoperative mortality.

Local institutional review board approval was obtained by all the participating centers. The study adhered to the Strengthening and Reporting of Observational Studies in Epidemiology (STROBE) guidelines^24^ and complied with the 1964 Helsinki Declaration and its later amendments.

### Definitions

For this study, IPMN-derived PDAC and not concomitant PDAC was confirmed by specialized local pathologists. These pancreas-specific pathologists at each high-volume center ensured that a direct association of the IPMN with the invasive component was present. The study excluded patients in whom no direct association (no clear pathologic connection) between the IPMN and invasive component was observed. However, in accordance with the international consensus guidelines, the combination of intestinal IPMN and colloid carcinoma inferred that the invasive component was IPMN-derived because concomitant PDAC exclusively shows tubular carcinoma.^25^

The T stage was evaluated using the size of the invasive component in accordance with the updated international consensus guidelines.^25^ The AJCC-TNM system eighth edition was evaluated for staging.^12^ Microscopic evidence of invasive cancer at or within 1 mm of the resection margin was defined as R1. Carbohydrate antigen 19-9 (CA19-9) was defined as non-secreter (<5 U/ml), normal (≥5 and ≤37 U/ml), or elevated (>37 U/ml).

Decisions regarding the use of adjuvant chemotherapy were institution dependent and most often made in a multidisciplinary setting. In general, resected IPMN-derived PDAC was treated like PanIN-derived PDAC given the absence of high-level evidence.

### Statistical Analysis

The patients were stratified according to the AJCC eighth-edition N stage (i.e., N0, N1, or N2). Continuous variables were summarized as mean ± standard deviation (SD) or as median with interquartile range (IQR) and compared using an analysis of variance (ANOVA) or Kruskal-Wallis test, respectively. Categorical variables were summarized as counts and percentages and compared using a chi-square or Fisher’s exact test when appropriate.

Overall survival (OS) was defined from the date of surgery to the date of death or last follow-up visit. Recurrence-free survival (RFS) was defined from the date of surgery to the date of death or recurrence, whichever came first, or last follow-up visit. Kaplan–Meier curves were performed to evaluate OS and RFS. Median OS and RFS values were calculated with 95 % confidence intervals (CIs). To compare Kaplan–Meier curves, a log-rank test was used.

Multivariable Cox-regression analysis was used to determine the prognostic value of N stage for OS and RFS. The variables initially included in the analysis were sex, age, CA19-9, T stage, N stage, resection margin, grade, histologic subtype, perineural invasion, lymphovascular invasion, and adjuvant chemotherapy. Backward selection was applied to select the combination of variables most predictive of OS and RFS. An additional multivariable analysis using the prognostic variables derived from the overall analysis was performed to assess the role of adjuvant chemotherapy and the prognostic importance of margin status in OS for patients with N0, N1, and N2 disease. Results are presented as hazard ratios (HRs) and corresponding 95 % CIs. Harrell’s C-statistic was reported to assess performance of the final model. A *p* value lower than 0.05 was used to define statistical significance.

To assess for a cutoff among the patients with node-positive disease, a lowest *p* value method using a log-rank test was used with OS as the outcome of interest. Multivariable Cox regression analysis was repeated, replacing AJCC N stage with the calculated optimal lymph node cutoff. Statistical analysis was performed with the R statistical software (version 4.2.3) using the “MaxStat,” “survminer,” “survival,” and “ggplot2” packages.

## Results

### Study Population

Of the 360 patients enrolled in the study, 56 % (*n* = 200) were male, 75 % (*n* = 269) were older than 65 years, and 61 % (*n* = 220) underwent pancreatoduodenectomy. The median number of harvested lymph nodes was 19 (IQR 14–27). The findings showed N0 disease present in 56 % (*n* = 201), N1 disease present in 24 % (*n* = 86), and N2 disease present in 20 % (*n* = 73) of the patients,. Further clinicopathologic data for the overall study population are summarized in Table S1.

With increasing N stage, worsening T stage (*p* < 0.001), poorer grade of differentiation (*p* = 0.006), more tubular than colloid histology (*p* < 0.001), and increasing prevalence of perineural (*p* < 0.001) and lymphovascular (*p* < 0.001) invasion were observed. In N1 and N2 disease, R1 margins also were more common (*p* < 0.001). Adjuvant chemotherapy was administered to 42 % (*n* = 81) of the patients with node-negative disease, whereas it was administered to 48 % (*n* = 40) of the patients with N1 disease, and 51 % (*n* = 36) of the patients with N2 disease (*p* = 0.332). These data are summarized in Table 1.

**Table 1:** Basic demographics and clinicopathologic information for all patients stratified by AJCC N stage with corresponding *p* values based on univariable comparisons

Variable	N0 (*n* = 201) *n* (%)	N1 (*n* = 86)* n* (%)	N2 (*n* = 73)* n* (%)	*p* Value
Male	119 (59)	44 (51)	37 (51)	0.293
Age >65 years	146 (73)	63 (73)	60 (82)	0.257
CA19-9				
Normal	62 (31)	13 (15)	15 (21)	
Elevated	64 (32)	37 (43)	28 (38)	0.086
Non-secreter	5 (3)	4 (5)	2 (3)	
Unknown	70 (35)	32 (37)	28 (38)	
Type of surgery				
Pancreatoduodenectomy	117 (58)	52 (60)	51 (70)	0.418
Distal Pancreatectomy	46 (23)	20 (23)	10 (14)	
Total Pancreatectomy	38 (19)	14 (16)	12 (16)	
T stage				
T1	110 (58)	15 (18)	9 (12)	
T2	47 (24)	49 (58)	39 (53)	**<0.001**
T3/4	41 (21)	20 (24)	25 (34)	
Unknown	3	2	0	
Median nodes harvested (IQR)	18 (14–26)	18 (13–28)	19 (14–28)	0.580
Median LNR (IQR)	0.00 (0–0)	0.07 (0.04–0.13)	0.30 (0.18–0.50)	**<0.001**
Grade of differentiation				
Poor	38 (21)	31 (36)	25 (36)	**0.006**
Unknown	3	0	3	
Tubular	100 (65)	69 (91)	56 (98)	**<0.001**
Unknown	24	8	11	
Perineural invasion	72 (38)	63 (74)	57 (80)	**<0.001**
Unknown	11	1	2	
Lymphovascular invasion	22 (12)	38 (44)	52 (72)	**<0.001**
R1-margin	20 (10)	17 (20)	27 (37)	**<0.001**
Adjuvant chemotherapy	81 (42)	40 (48)	36 (51)	0.332
Unknown	8	3	3	

Bold values indicate statistical significance (*p* < 0.005)

*AJCC* American Joint Committee on Cancer; *CA19-9* carbohydrate antigen 19-9; *IQR* interquartile range; *LNR* lymph node ratio

### OS and RFS

**Fig. 1:**
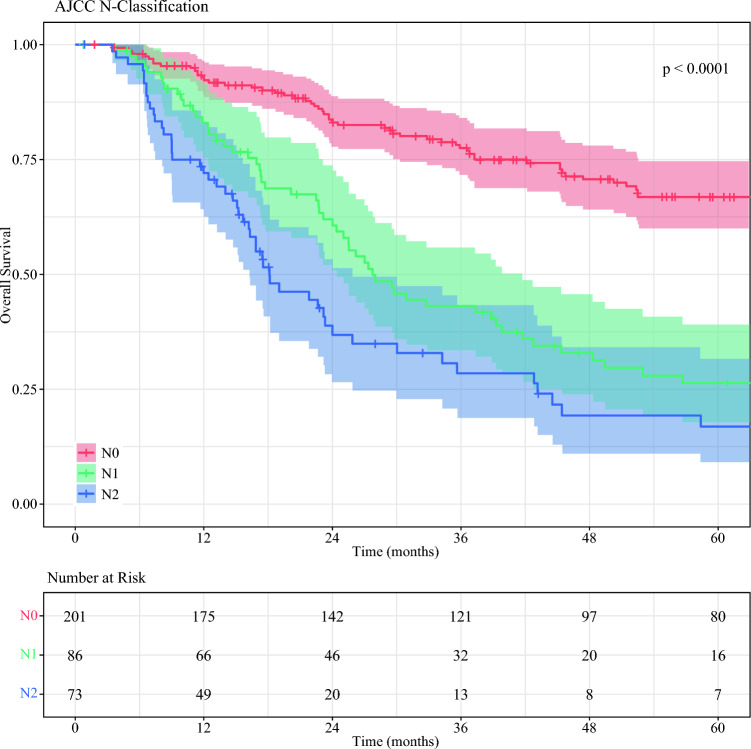
Kaplan–Meier curves comparing overall survival stratified by American Joint Committee on Cancer (AJCC) nodal stage.

The median follow-up period for the surviving patients was 41.1 months (IQR 17.5–74.8 months). Using the AJCC eighth-edition N-staging system, the median OS was 98.3 months (95 % CI 82.8–122.0 months) for the patients with N0 disease, 27.8 months (95 % CI 24.4–41.7 months) for the patients with N1 disease, and 18.1 months (95 % CI 16.2–25.9 months) for the patients with N2 disease (*p* < 0.001). Pairwise comparison showed that the patients with N0 disease had better OS than those with N1 (*p* < 0.001) or N2 (*p* < 0.001) disease. The patients with N2 disease had worse OS than those with N1 disease (*p* = 0.023; Fig. 1)

**Fig. 2:**
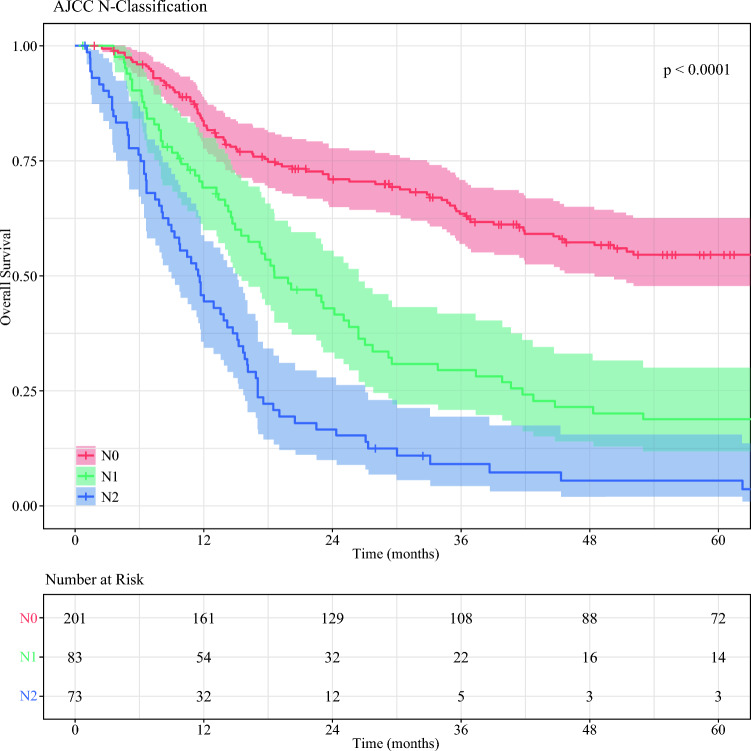
Kaplan–Meier curves comparing recurrence-free survival stratified by American Joint Committee on Cancer (AJCC) nodal stage.

The median RFS was 72.4 months (95 % CI 48.4–93.9 months) for the patients with N0 disease, 18.6 months (95 % CI 15.4–26.4 months) for the patients with N1 disease, and 11.6 months (95 % CI 9.0–15.1 months) for the patients with N2 disease (*p* < 0.001). Pairwise comparison showed that the patients with N0 disease had better RFS than those with N1 (*p* < 0.001) or N2 disease (*p* < 0.001). The patients with N2 disease had worse RFS than those with N1 disease (*p* < 0.001) (Fig. 2).

### Cox Regression for OS and RFS

**Fig. 3:**
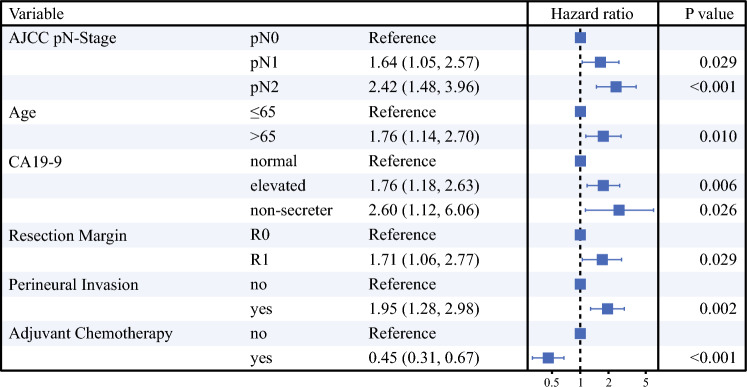
Multivariable Cox regression analysis with backward selection to identify the combination of variables best predictive for overall survival and to determine the prognostic value of the American Joint Committee on Cancer (AJCC) N-stage classification.

Multivariable Cox regression analysis showed that higher AJCC pN-stage classification was associated with worse OS (N1 [HR 1.64; 95 % CI 1.05–2.57; *p* = 0.029], N2 [HR 2.42; 95 % CI 1.48–3.96; *p* < 0.001]). Other variables included in the model were age, CA19-9, margin, perineural invasion, and adjuvant chemotherapy. Harrell’s C-statistic for OS in the final model was 0.75. These results are summarized in Fig. 3.

**Fig. 4:**
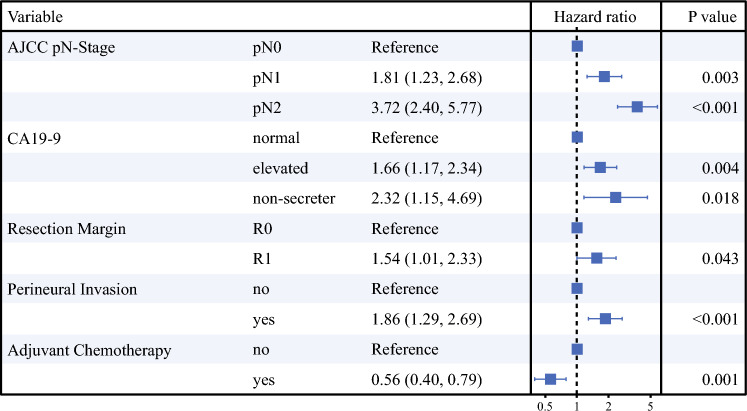
Multivariable Cox-regression analysis with backward selection to identify the combination of variables best predictive for recurrence free survival and to determine the prognostic value of the American Joint Committee on Cancer (AJCC) N-stage classification.

Multivariable Cox regression analysis showed that increasing AJCC pN-stage classification was significantly associated with worse RFS (N1 [HR 1.81; 95 % CI 1.23–2.68; *p* = 0.003], N2 [HR 3.72; 95 % CI 2.40–5.77; *p* < 0.001]). Other variables included in the model were CA19-9, margin, perineural invasion, and adjuvant chemotherapy. Harrell’s C-statistic for RFS in the final model was 0.74. These results are summarized in Fig. 4.

### Impact of Adjuvant Chemotherapy and Margin Status on OS in Each Nodal Stage

For the N0 patients, worse OS was associated with age older than 65 years (HR 3.01; 95 % CI 1.52–5.96; *p* = 0.002), elevated CA19-9 (HR 1.91; 95 % CI 1.11–3.29; *p* = 0.02), R1 margin (HR 2.51; 95 % CI 1.16–5.40; *p* = 0.019), and perineural invasion (HR 1.79; 95 % CI 1.03–3.12; *p* = 0.039), whereas adjuvant chemotherapy was not (*p* = 0.160). For the N1 patients, margin status was not significantly associated with OS (HR 2.45; 95 % CI 0.94–0.42; *p* = 0.070), whereas adjuvant chemotherapy was associated with improved OS (HR 0.46; 95 % CI 0.23–0.94; *p* = 0.033). For the N2 patients, again, margin status was not associated with OS (HR 0.78; 95 % CI 0.32–1.91; *p* = 0.588), whereas adjuvant chemotherapy was associated with improved OS (HR 0.25; 95 % CI 0.11–0.54; *p* = 0.033).

### Cutoffs for Node-Positive Disease

Potential lymph node positivity cutoffs with significant discriminatory ability were two, three, four, five, or six positive lymph nodes (all *p* < 0.05). The lowest *p* value was noted at a cutoff of five or more positive lymph nodes. In the Cox regression analysis for OS using the five-lymph-node cutoff, N-stage remained associated with worse survival (N1 [HR 1.59; 95 % CI 1.03–2.44; *p* = 0.035], N2 [HR 3.00; 95 % CI 1.79–5.05; *p* < 0.001]). Other variables included in the model were age, CA19-9, margin, perineural invasion, and adjuvant chemotherapy. Harrell’s C-statistic for the final model including the five-lymph-node cutoff was 0.76. These results are summarized in Fig. S1.

In the Cox regression analysis for RFS using the five-lymph-node cutoff, N stage remained associated with worse survival (N1 [HR 1.90; 95 % CI 1.32–2.76; *p* < 0.001], N2 [HR 4.50; 95 % CI 2.80–7.23; *p* < 0.001]). Other variables included in the model were CA19-9, margin, perineural invasion, and adjuvant chemotherapy. Harrell’s C-statistic for the final model including the five-lymph-node cutoff model was 0.74. These results are summarized in Fig. S2.

## Discussion

The current AJCC staging system for pancreatic cancer is derived from studies and validations mostly composed of PanIN-derived PDAC. Due to a lack of evidence-based studies for IPMN-derived PDAC, its management and prognostication often are based on our more developed and comprehensive understanding of PanIN-derived PDAC.

This study leveraged a multi-institutional experience, investigated the role of N stage in IPMN-derived PDAC, and validated the current AJCC pN stage. We observed that advancing N stage was associated with worsening histopathologic features as well as OS and RFS. We also found that the current eighth-edition AJCC N-staging is valid and provides robust discrimination for IPMN-derived PDAC.

In our study, we found that advancing N stage was associated with worsening histopathologic features, mostly in a stepwise manner. These factors included worsening T stage, grade of differentiation, tubular histologic subtype, and perineural and lymphovascular invasion. This highlights that advanced nodal disease is associated with worse disease biology in IPMN-derived PDAC. A similar trend has been described in PanIN-derived PDAC as well.^26,27^

Interestingly, the study additionally observed that incidence of an R1 resection margin also was associated with worse nodal disease. This may be partially explained by the trend seen in worsening T stage and more locally aggressive disease. This finding also is consistent with studies investigating PanIN-derived PDAC.^26,28^ Interestingly, the use of adjuvant chemotherapy did not significantly vary based on N stage, although multiple retrospective studies have observed nodal disease as the best clinicopathologic factor in determining the controversial need for adjuvant chemotherapy.^29,30^

Recently, Margonis et al.^23^ evaluated the performance of the seventh and eighth editions of the AJCC staging systems for 275 patients. In their analysis, they found no significant difference in OS in a comparison of patients with N1 and N2 disease (*p* = 0.497) per the AJCC eighth-edition recommendation. Accordingly, they recommended regrouping patients with N1 and N2 disease, which is concordant with the AJCC seventh-edition recommendation.^13,23^ However, our more strongly powered study found a significant difference between these two cohorts, thus successfully validating the AJCC eighth-edition N stage classification. Furthermore, our study found multiple potential cutoffs for significant discrimination in positive nodal stage prognostication. The optimal cutoff was found to be five lymph nodes or more for N2 disease, which is just one node greater than the current AJCC N2-stage definition recommends.^12^ However, the prognostic model performance was comparable with both cutoffs. These data thus support the use of the currently recommended AJCC eighth edition in IPMN-derived PDAC.

Interestingly, the median OS was 98.3 months for the patients with N0 disease, 27.8 months for the patients with N1 disease, and 18.1 months for the patients with N2 disease. The median RFS values for these cohorts were respectively 72.4, 18.6, and 11.6 months. Whereas the patients without nodal disease were observed to have excellent outcomes, the patients with nodal disease had poorer outcomes, comparable with the OS and RFS observed for patients with resected PanIN-derived PDAC.^3^ This could potentially explain why adjuvant chemotherapy was found to be associated with improved survival for the patients with N1 or N2 disease and not for those with N0 disease, with similar observations for all the patients with PanIN-derived PDAC. This suggests that when patients with IPMN-derived PDAC experience nodal disease, they behave like patients with PanIN-derived PDAC. The biologic underpinnings and clinical implications of this finding need to be explored further.

Nodal disease has been strongly implicated in outcomes for IPMN-derived PDAC in multiple studies.^20,22,31,32^ The multivariable Cox regression found the AJCC N stage classification to be a significant predictor in modeling prognosis for both OS and RFS. Age, preoperative CA19-9 level, margin status, perineural invasion, and use of adjuvant chemotherapy also emerged as significant prognostic factors. Thus, these data suggest that current the AJCC eighth edition for N-staging is a robust factor that should be used in patient stratification.

In N-stage sub-analysis, we observed that adjuvant chemotherapy was associated with improved outcomes only for patients with nodal disease, as previously described.^29,33,34^ Additionally, an R1 margin status appears to be associated with poorer survival only for patients with N0 disease, whereas with increasing nodal status, an R0 margin status appears to be less clinically relevant.

Several limitations of the current study should be acknowledged. First, the study included patients who underwent resection over a long study period during which clinical practice and chemotherapy regimens changed. However, this was done to ensure a large study population given the relative infrequency of resected IPMN-derived PDAC. Second, the retrospective nature of this study led to some missing data that may have influenced results. Finally, different surgical and pathologic assessment practices may have existed across participating centers.

Future prospective studies should investigate the role of lymph node station and nodal staging, and centralization of pathologic evaluation may mitigate potential differences in pathologic reporting practices. Nevertheless, the current study represents the largest investigation validating the AJCC eighth edition for N-staging of IPMN-derived PDAC.

## Conclusion

This international multi-institutional study examined the role of positive lymph nodes and validated the current AJCC eighth-edition classification for nodal disease in IPMN-derived PDAC. Advancing nodal disease burden is associated with worse tumor biology and emerges as a robust predictor of OS and RFS. The eighth edition of the AJCC N-stage classification is prognostic for both OS and RFS and therefore valid when applied in IPMN-derived PDAC.

## Supplementary Information

Below is the link to the electronic supplementary material.

Supplementary file1 (DOCX 2148 kb)
