# Supplementary material for: Evaluation of AJCC Nodal Staging for Intraductal Papillary Mucinous Neoplasm-Derived Pancreatic Ductal Adenocarcinoma
Source: Ann Surg Oncol. 2024 Sep 16;31(13):8712–20. doi: 10.1245/s10434-024-16055-5 (PMC11549140; doi:10.1245/s10434-024-16055-5)
Supplement: Supplementary file 1 — Supplementary file1 (DOCX 2148 kb) [file 10434_2024_16055_MOESM1_ESM.docx]

**SUPPLEMENT**

**Table S1.** Baseline demographics and clinicopathologic information of overall study population.

| **Variable** | **n=360** |
| --- | --- |
| **Male, n (%)** | 200 (56%) |
| **Age > 65, n (%)** | 269 (75%) |
| **CA19-9, n (%)** |  |
| normal | 90 (39%) |
| elevated | 129 (56%) |
| non-secreter | 11 (5%) |
| Unknown | 130 |
| **Type of Surgery** |  |
| PD | 220 (61%) |
| DP | 76 (21%) |
| TP | 64 (18%) |
| **T-stage, n (%)** |  |
| T1 | 134 (38%) |
| T2 | 135 (38%) |
| T3/4 | 86 (24%) |
| Unknown | 5 |
| **Poor Grade of Differentiation, n (%)** | 94 (28%) |
| Unknown | 22 |
| **Tubular, n (%)** | 225 (79%) |
| Unknown | 74 |
| **Nodes Harvested, Median (IQR)** | 19 (14-27) |
| **Lymph Node Ratio, Mean (IQR)** | 0.0 (0.0-0.13) |
| **AJCC N-stage, n (%)** |  |
| N0 | 201 (56%) |
| N1 | 86 (24%) |
| N2 | 73 (20%) |
| **R1-margin, n (%)** | 64 (18%) |
| **Perineural Invasion, n (%)** | 192 (55%) |
| Unknown | 14 |
| **Lymphovascular Invasion, n (%)** | 112(32%) |
| Unknown | 11 |
| **Adjuvant Chemotherapy, n (%)** | 157 (45%) |
| Unknown | 14 |

**Figure S1.** Multivariable Cox-regression analysis with backwards selection to identify the combination of variables best predictive for overall survival and to determine the prognostic value N-stage using the five of more lymph node cut-off.


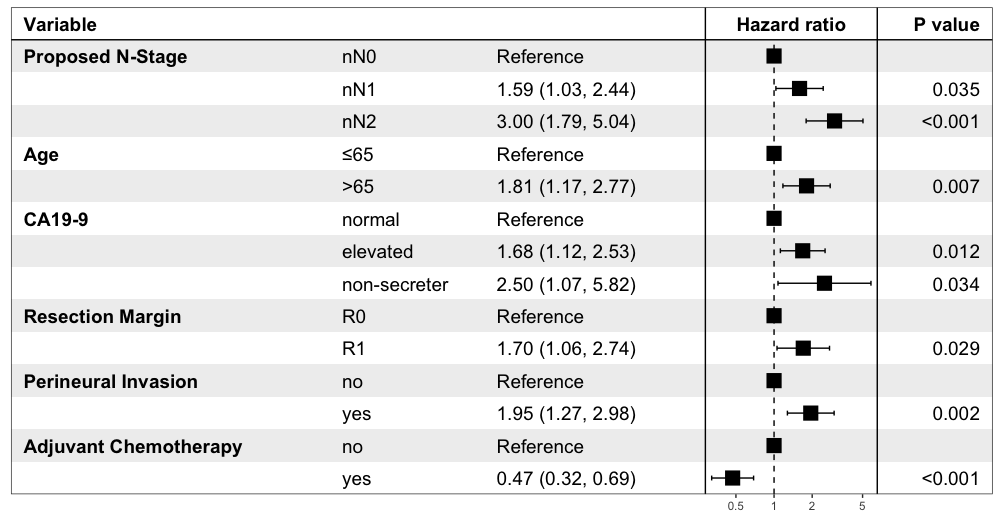


**Figure S2.** Multivariable Cox-regression analysis with backwards selection to identify the combination of variables best predictive for recurrence free survival and to determine the prognostic value N-stage using the five of more lymph node cut-off.

**
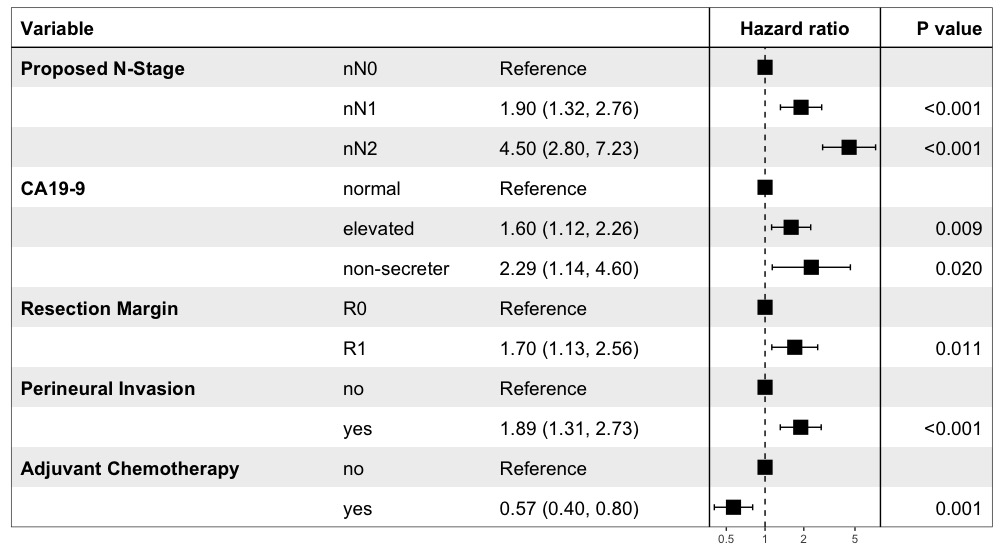
**
